# Ten Years of Experience Support Pharmacogenetic Testing to Guide Individualized Drug Therapy

**DOI:** 10.3390/pharmaceutics14010160

**Published:** 2022-01-11

**Authors:** María Celsa Peña-Martín, Belén García-Berrocal, Almudena Sánchez-Martín, Elena Marcos-Vadillo, María Jesús García-Salgado, Santiago Sánchez, Carolina Lorenzo, David González-Parra, Francisco Sans, Manuel Franco, Andrea Gaedigk, María José Mateos-Sexmero, Catalina Sanz, María Isidoro-García

**Affiliations:** 1 Department of Clinical Biochemistry, University Hospital of Salamanca, 37007 Salamanca, Spain; mariacelsa@usal.es (M.C.P.-M.); mbgarcia@saludcastillayleon.es (B.G.-B.); emarcosv@saludcastillayleon.es (E.M.-V.); mjgarciasal@saludcastillayleon.es (M.J.G.-S.); 2 Department of Pharmacy, University Hospital of Salamanca, 37007 Salamanca, Spain; almuweb06@gmail.com; 3 Department of Psychiatry, University Hospital of Salamanca, 37007 Salamanca, Spain; sisanchez@saludcastillayleon.es (S.S.); carolinalorenzo@usal.es (C.L.); dgonzalezp@saludcastillayleon.es (D.G.-P.); 4 Psychiatry and Mental Health Department, Healthcare Complex of Zamora, 49022 Zamora, Spain; fjsans@saludcastillayleon.es (F.S.); mfrancom@saludcastillayleon.es (M.F.); 5 Psychiatry and Mental Health Department, University Rio Hortega Hospital, 47012 Valladolid, Spain; 6 Biomedicine Research Institute (IBSAL), PETRA Department, 37007 Salamanca, Spain; 7 Division of Clinical Pharmacology, Toxicology & Therapeutic Innovation, Children’s Mercy, Kansas City, MO 64108, USA; agaedigk@cmh.edu; 8 Psychiatry and Mental Health Department, University Hospital of Valladolid, 47003 Valladolid, Spain; mmateoss@saludcastillayleon.es; 9 Department of Microbiology and Genetics, University of Salamanca, IBSAL, 37007 Salamanca, Spain; catsof@usal.es; 10 Department of Medicine, University of Salamanca, IBSAL, 37007 Salamanca, Spain

**Keywords:** pharmacogenetics, polypharmacy, precision medicine

## Abstract

Precision medicine utilizing the genetic information of genes involved in the metabolism and disposition of drugs can not only improve drug efficacy but also prevent or minimize adverse events. Polypharmacy is common among multimorbid patients and is associated with increased adverse events. One of the main objectives in health care is safe and efficacious drug therapy, which is directly correlated to the individual response to treatment. Precision medicine can increase drug safety in many scenarios, including polypharmacy. In this report, we share our experience utilizing precision medicine over the past ten years. Based on our experience using pharmacogenetic (PGx)-informed prescribing, we implemented a five-step precision medicine protocol (5SPM) that includes the assessment of the biological–clinical characteristics of the patient, current and past prescription history, and the patient’s PGx test results. To illustrate our approach, we present cases highlighting the clinical relevance of precision medicine with a focus on patients with a complex history and polypharmacy.

## 1. Introduction

One of the main objectives in health care is a safe and efficacious pharmacological intervention. However, drug response is highly variable and may depend on genetic variation, among other factors. The field of pharmacogenetics and genomics studies the impact of genes and genetic variation on drug absorption, distribution, metabolism, excretion (also referred to ADME), as well as the response of an individual (or lack thereof) to a given drug or combination of drugs [1]. Thus, precision medicine should become the standard of care, replacing “one size fits all” approaches when appropriate. Pharmacogenetic testing, ideally obtained before treatment initiation, can be a powerful tool to enhance patient care and treatment outcomes [2].

Individual drug response is based on the pharmacokinetic and pharmacodynamic properties of the therapeutic agent(s), among others. Pharmacokinetics (PK) describes the processes and fate of the drug, taking into account absorption, distribution, metabolism, and excretion. In contrast, pharmacodynamics (PD) describes the biochemical and physiologic effects of the body in response to a drug or drugs; drug response often includes drug receptors. Other factors impacting PK and PD include compliance (taking the drug(s) as instructed), drug properties (formulation, pharmacologically active metabolites), and bioavailability (the fraction of an administered dose of the drug that reaches the therapeutic target).

Among the most relevant pharmacogenes affecting PK are members of the cytochrome P450 (CYP450) family. Enzymes encoded by the CYP1, CYP2, CYP3, and CYP4 families are significant contributors to the Phase I biotransformation of drugs and xenobiotics [3,4,5,6]; more than 75% of drugs undergoing hepatic clearance are indeed metabolized by CYPs of these families [7,8,9]. There is mounting evidence that genetic variation of CYP genes contributes to lack of efficacy, drug interactions, and adverse events [9,10,11,12]. The diverse combination of the different mechanisms and factors, such as genetic variation, influence the activity of each CYP enzyme [13]. The major CYP isoforms responsible for the metabolism of many drugs include CYP1A2, CYP2B6, CYP2C8, CYP2C9, CYP2C19, CYP2D6, CYP2E1, and CYP3A4/A5. CYP3A4 is the predominant isoform of the CYP family, usually responsible for the metabolism of approximately 37% of drugs, followed by CYP2C9 (17%), CYP2D6 (15%), CYP2C19 (10%), CYP1A2 (9%), CYP2C8 (6%) and CYP2B6 (4%) [6,14] Although CYP3A4 contributes to the metabolism of numerous drugs, genetic variation is not a major contributing factor for highly variable activity [15]. In contrast, CYP2D6 is highly polymorphic with over 140 different star alleles variants described to date (cataloged by the Pharmacogene Variation Consortium, available online: https://www.pharmvar.org/ (accessed on 26 January 2020)) [16,17,18], causing a wide range of activity [19]. Although the Clinical Pharmacogenetics Consortium (CPIC) published recommendations for the translation of genotype phenotype is not standardized across clinical laboratories [19]. It is necessary to take into account that the pharmacogenetic variations are different in regard to the different populations around the world [20].

Therefore, determining a patient’s metabolic profile for one or multiple ADME genes can guide drug selection and dose to avoid adverse events related to the drug’s metabolism [21,22]. Phase II enzymes such as UGTs and drug transporters such as ABCB1 (MDR1) also contribute to drug metabolism and response [23]. Another transporter of importance is SLCO1B1, which is associated with plasma concentrations of certain statins (HMG-CoA reductase inhibitors), increasingly prescribed to decrease the risk of cardiovascular diseases [24].

With aging populations, worldwide polypharmacy is becoming the rule rather than the exception for the elderly and comorbid adult patients. Concerns about polypharmacy include adverse events due to drug–drug interactions and pose a financial burden on health systems. It has been shown that both increase with the number of drugs prescribed for a patient [25,26,27].

Taking all relevant patient information into account, including PGx test results, will allow the clinician to tailor drug therapy, i.e., the choice of drug(s) and dose, for each patient, and practice what is commonly referred to as precision medicine. We have developed a 5-step precision medicine (5SPM) polypharmacy protocol and applied this approach in clinical practice. In this report, we summarize our ten-year experience utilizing PGx testing and practicing precision medicine.

## 2. Materials and Methods

### 2.1. Subjects and Study Design

All patients who underwent testing in the Pharmacogenetic Unit of the University Hospital of Salamanca over 10 years (2007 to 2016) were included, regardless of their origin. All methods were carried out following relevant guidelines and regulations. Written informed consent was obtained from all patients according to the recommendations of the Ethical Committee of the University Hospital of Salamanca. This committee approved the study with the Ref Cod. CEIm PI 2020 12 642.

Study patients were classified into two groups:

- Monotherapy: a patient’s PGx test was requested for a specific gene.
- Polytherapy: patients on polypharmacy. A model designed to analyze polymedicated patients, called 5SPM, was applied to this patient group.

### 2.2. 5-Step Precision Medicine Model

#### 2.2.1. Step 1: Clinical, Epidemiological and Therapeutic Data Collection

Data collection included each patient’s medical condition(s), current prescriptions (classified by the anatomical, therapeutic, chemical (ATC) classification system), and therapeutic response. The number of prescribed drugs ranged from 2 to 18 for the polytherapy group. The reason for referral to the precision medical unit for PGx testing (e.g., adverse effects, therapeutic failure) was recorded, as well as patient age, gender, and medication history.

Drugs were classified using the ATC system according to the organ or system on which they act and their therapeutic, pharmacological, and chemical properties per the World Health Organization Collaborating Centre for Drug Statistics Methodology (WHOCC) [28].

#### 2.2.2. Step 2: Predictions of Drug–Drug Interactions and Pharmacokinetic Specific Pathways

Publicly available resources, including the Pharmacogenomic Knowledgebase (PharmGKB) [29] PubMed-NCBI [30], SuperCYP [31], and the Pharmacogene Variation (PharmVar) Consortium [16,32], were used to analyze drug–gene and drug–drug interaction of the specific drugs prescribed to each patient.

#### 2.2.3. Step 3: Pharmacogenetic Analysis of Selected Genes

PGx testing was performed in probe-based assays using the LightCycler platform (Roche Diagnostics), the AmpliChip CYP450 Test (Roche Molecular Diagnostics, Pleasanton, CA, USA) [33], and the Autogenomics platform (Carlsbad, CA, USA) [34]. As previously reported [35], PGx laboratory testing was performed following the directives of the European Molecular Genetics Management Network. Quality norms were applied following the UNE-EN-ISO 15189 Normative in the Accredited Section of Molecular Genetics and Pharmacogenetics laboratory of the University Hospital in Salamanca. The normative included preanalytical, analytical and post-analytical control, qualification of personal, and internal and external validity.

Selected *CYP2D6* no-call results were further characterized in the Division of Clinical Pharmacology, Toxicology & Therapeutic Innovation laboratory at Children’s Mercy Kansas City using a combination of approaches including long-range (XL) PCR, Sanger sequencing and quantitative copy number analysis [36,37,38].

Genotype was translated into phenotype per the CYP2D6 Diplotype-Phenotype Table available through PharmGKB (available online: https://www.pharmgkb.org/page/pgxGeneRef (accessed on 3 June 2018)).

#### 2.2.4. Step 4: Rationalized PGx-Guided Adjustments of Drug Therapy

The drugs prescribed and their dose were revised or changed based on a patient’s predicted phenotype and the potential drug–drug interactions of the drugs prescribed. The objectives were to identify which drugs were not efficiently metabolized, which drugs were mainly metabolized by the same enzyme, and which drugs were inhibitors causing the patient to have phenotype different from that predicted by genotype (also known as phenocopy) with the goal to decrease potential harmful drug interactions. The patient’s prescription was modified accordingly.

#### 2.2.5. Step 5: Assessment of the Intervention and Model Reevaluation

The model was continuously evaluated by analyzing patient outcome based on the changes made following PGx testing. Clinicians followed the outcome of each patient and provided feedback regarding the intervention.

### 2.3. Statistical Analysis

Descriptive statistics was used to determine central tendency and dispersion. The normality of the distribution was assessed using the Kolmogorov–Smirnov test. Bivariate analysis for qualitative variables was carried out by χ2, Fisher exact test, and Montecarlo test. Bivariate analysis for quantitative and qualitative variables was carried out by ANOVA. The equality of variances was ensured using the Levene test. All statistical analyses were performed using SPSS version 17.0 (Chicago, IL, USA).

## 3. Results

A total of 1540 patients were studied in the Pharmacogenetics and Precision Medicine Unit of the University Hospital of Salamanca. A total of 1340 patients were referred to the unit to analyze the response to a specific drug, however, as shown in Table 1, only 210 patients were qualified for the 5SPM model due to experiencing adverse effects, intolerance to treatment, partial response, or therapeutic failure in a polypharmacy regimen, as we can observe in Table 1.

**Figure 1:**
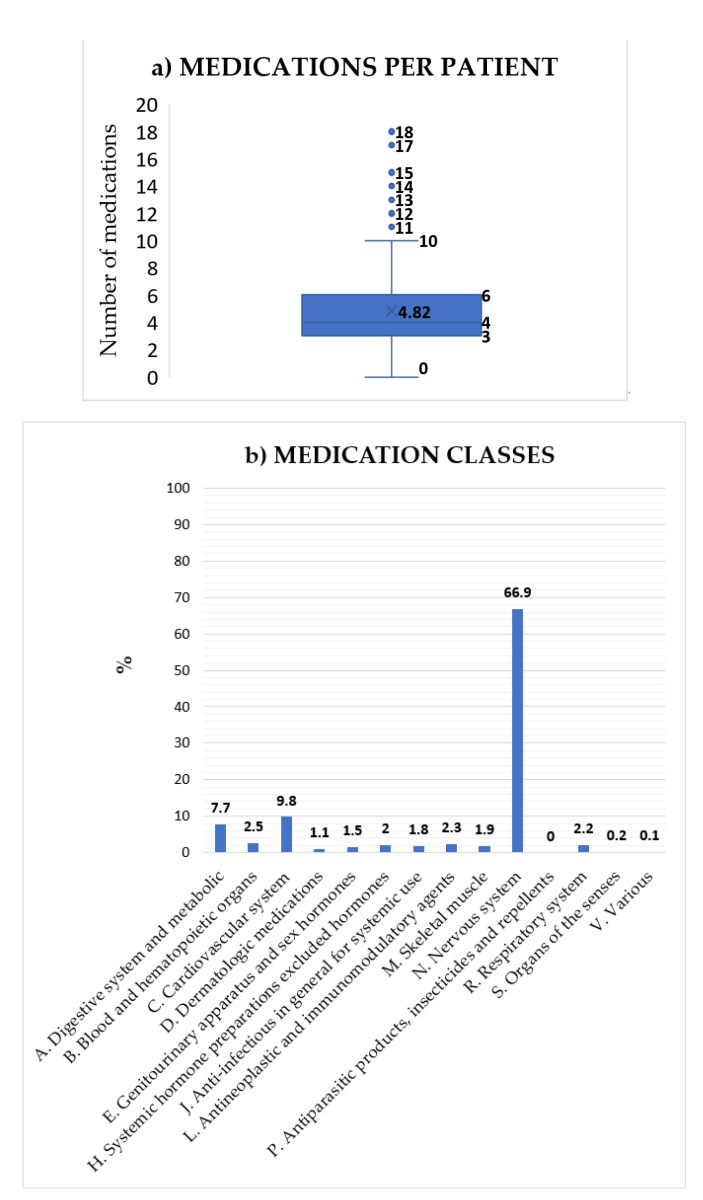
Medication-use data: (**a**) number of medications per patient (mean); (**b**) type of medication.

Table 1 and Supplementary Figures S1 and S2 summarizes patient demographics, as well as the reasons for genetic analysis and clinical services. Figure 1 includes selected data of the analyzed drugs and the classification of these medications according to the ATC nomenclature, highlighting more than 60% of the central nervous system group.

### 3.1. Clinical Data Collection

Half of the patients were female (49.5%). The mean age is 43.9 ± 18.1 vs. 47.1 ± 18.8 in males. Supplementary Figure S3 shows a box plot with the age distribution. No statistically significant differences were observed between both groups according to age (Fisher’s *p*-value = 0.21). Fifteen % of the patients were older than 65 years of age. Of the enrolled patients, 92.5% had neuropsychiatric disorders, mainly psychosis, depression, and bipolar disorders representing 66.74% of the medications, followed by patients treated for cardiovascular conditions (22.5% of patients representing 9.8% of the medications).

Only in 1% of cases was PGx analysis performed before the initial prescription. Nearly half of the patients (47.1%) were referred to the unit for adverse events or intolerance, and 19.0% were referred owing to partial response or therapeutic failure. Therapeutic failure was observed more often in men and adverse events in women, although these observations were not statistically significant (Fisher’s *p*-value = 0.26).

Psychiatric patients most often complained that the drug did not help (83.9%), contrasting patients seen in the allergy department, which reports that 75% had adverse events (Fisher’s *p*-value < 0.001).

### 3.2. Pharmacological Interactions

A total of 956 prescriptions were recorded for these patients, with a mean of 5 drugs per patient (range 1 to 18 drugs); 45% of the patients had prescriptions for 5 or more drugs. Potential drug–gene (Table 2) and drug–drug (Table 3) interactions were analyzed. For drug–gene interactions, patients predicted to have poor, intermediate, or ultrarapid phenotypes were selected, and may therefore have a higher risk to present with toxicity or treatment failure due to drug–gene interaction(s). A total of 728 potential drug–gene interactions were identified. From these interactions, we selected 25 medications which were most commonly prescribed (Table 2), and 24 of them had the highest number of interactions (Table 3). For the analysis of drug–drug interactions, route of metabolism (same or different routes) and whether they act as inhibitors, substrates or inducers was taken into account. A total of 2030 possible drug-drug interactions were determined. Supplementary Figure S4 summarizes the top 10 drug–gene and drug–drug interactions.

**Figure 2:**
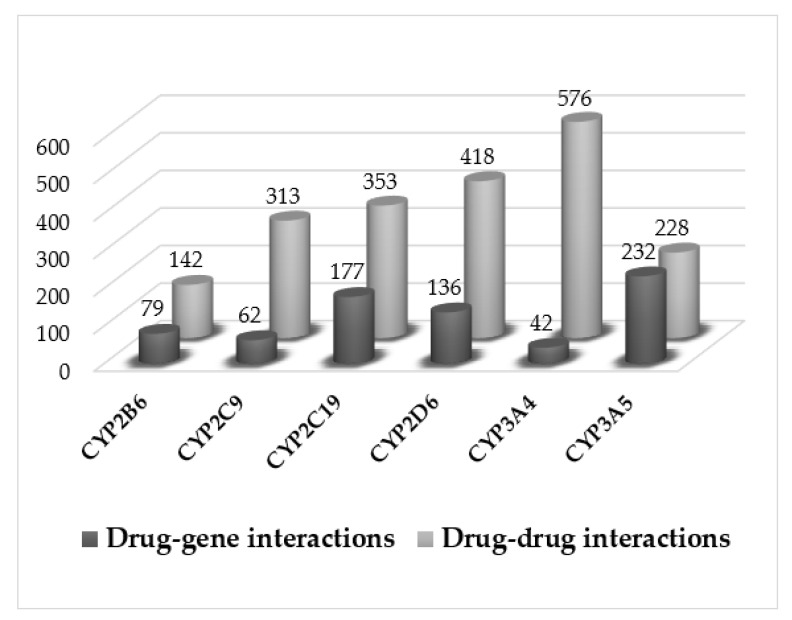
Potential drug–gene and drug–drug interactions counts in the study population.

Finally, as shown in Figure 2, drug–gene interactions were compared with drug–drug interactions for each CYP isoenzyme. This allowed us to determine the percentage of drug–drug interactions that could be avoided according to genotype. For CYP3A4, the majority of individuals had genotypes predicting normal metabolism. However, for other enzymes such as CYP3A5 or CYP2C19, genotype played a considerably more significant role suggesting that the inclusion of genotype information could avoid a high percentage of interactions (Fisher’s *p*-value < 0.001).

### 3.3. Pharmacogenetic Analysis

A total of 210 patients were genotyped for genetic variants, including 10,400 alleles corresponding to *CYP1A2, CYP2B6, CYP2C9, CYP2C19, CYP2D6, CYP3A4, CYP3A5,* and *ABCB1* as detailed in in Table 4.

**Figure 3:**
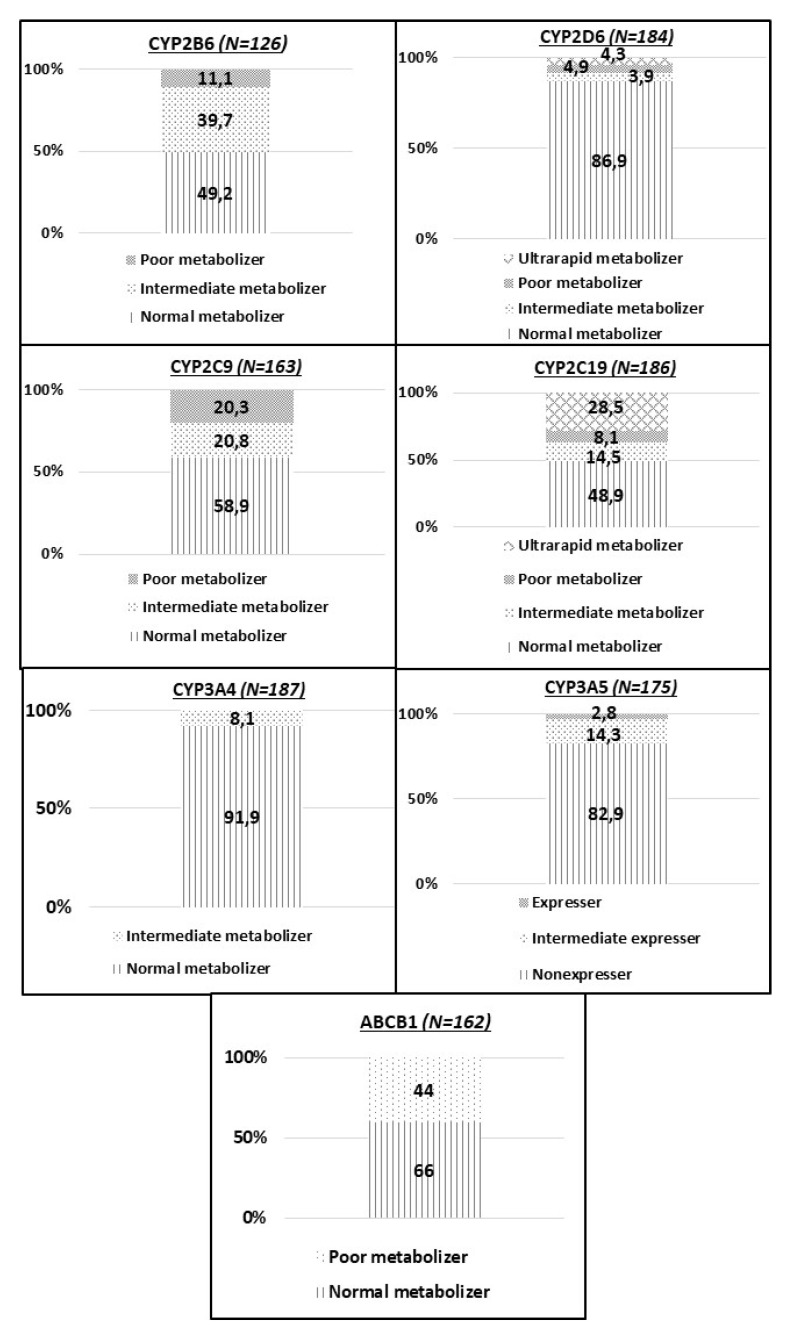
Distribution of evaluated cytochrome P-450 (CYP) metabolic phenotypes in the study population.

Figure 3 summarizes the genotype and phenotype information of the patients. We like to highlight that PGx testing and analysis were performed individually, i.e., in a personalized manner for each patient based on their genotype and drug treatment. Concerning *ABCB1*, up to 66% of patients carried at least one T allele that has been associated with decreased transporter function.

Significant differences were observed regarding the phenotype and the indication of the study. For instance, 39.4% of patients who were intermediate metabolizers for CYP2C9, Fisher’s *p*-value = 0.020, were included in the study due to adverse event reports vs. 16.7% due to intolerance to treatment.

Regarding CYP2D6, the most common phenotype was normal metabolizers (86.9%). On the other side, allele frequencies associated with intermediate and poor metabolism were 0.35.

There was also an increase of the enzyme activity, such as CYP2C19 allele *17, corresponding to increased activity of the enzyme (allelic frequency 0.17 and genotypic frequency 0.03) and CYP2D6 (4.4% of increased enzyme activity corresponding to different alleles that causes increased enzyme activity).

### 3.4. Identification of Novel Allelic Variants

Some patients had no-calls when genotype was tested on the Roche AmpliChip. Further characterization of selected cases by Sanger sequencing revealed three *CYP2D6* haplotypes, which were not recognized by the AmpliChip software. Among them were theno function CYP2D6*31 allele [36], and the CYP2D6*9x2 gene duplication [37]. Interestingly, this duplication is less frequent in Salamanca than in other regions worldwide. [37]. Furthermore, one patient revealed a complex genotype, CYP2D6*68 + *4/*77 + *2 with tandem arrangements and hybrid genes on both alleles which likely interfered with the AmpliChip test [38].

### 3.5. Clinical Results

The integration of PGx into practice allowed us to gain valuable information regarding the therapeutic management of patients. An example of the clinical application of the model has previously been described by Isidoro et al. [39].

Psychiatric Patients

To demonstrate our approach, we present here the results for psychiatric patients. These were as follows: 40% of psychiatric patients had genotypes predicting altered enzymatic activity that could impact pharmacologic response. Specifically, 92% of patients had gene variants affecting the metabolism of CYP2D6 substrates. Among bipolar patients, all having a poor metabolizer phenotype showed maniac switching when CYP2D6 substrates, such as selective serotonin reuptake inhibitors, were prescribed [37].

Among patients with eating disorders, 64% of anorexic patients suffered from adverse events; each of these patients had a genotype that affected the metabolism of some of the prescribed medications and clinical symptoms improved for 93.8% of the patients (i.e., gained weight) after undergoing the application of precision medicine.

### 3.6. Economic Implications

Considerable cost reductions were observed after the application of PGx testing in our patients. For example, for a representative group of patients with infectious diseases, up to €8182 were saved for each patient with CMV treated with interferon, or €43,549/year for HIV patients treated with efavirenz [40] by genotyping *IL28* or *CYP2B6,* respectively. Applying a theoretical model allowed us to infer up to €587/patient of avoided hospital costs [26]. This model was developed in 2014 in the University Hospital of Salamanca and allowed us to infer considerable cost reductions if the PGx testing took place before the outcoming of adverse effects. It also took into account the quality of the age expectancy [26].

An economic impact of the model in psychotic patients has been previously reported [41]. In this study, we achieved a reduction in direct costs, including both hospitalization and pharmacotherapy, as well as a reduction in total costs in 67% of patients who underwent the application of this pharmacogenetic approach.

In addition, PGx testing of a gene panel was up to €500 and results were useful to inform current therapy and have the potential to guide future medication needs. Thus, PGx testing is cost-effective over a patient’s lifetime.

## 4. Discussion

Polypharmacy is unavoidable for many patients, especially the elderly or those suffering from complex diseases or conditions. It has been shown that the more physicians are involved in treating a patient, the higher the risk of adverse events [39]. In addition, patients with an unfavorable pharmacological response tend to be treated with increasing doses or numbers of drugs, which negatively impacts the patient and adds to increased costs. Concerns about polypharmacy include adverse events, interactions, and costs, that increase with the number of drugs. Taken together, this points to an urgent need for personal medicine models that consider drug interactions. As demonstrated by our model, the integration of PGx and careful evaluation of drug–drug and drug–gene interactions has the potential of benefitting a large number of patients.

As polypharmacy increases with age, there is an additional risk of experiencing adverse events to pharmacological drugs after age 65 [26]. However, in our study cohort, only 15% of the patients with polypharmacy were older than 65, strongly suggesting that this is not solely an age-related problem but also affects many younger patients.

In this investigation, most patients were studied after receiving a prescription, which had the advantage of assessing relationships with the clinical response. Thus, potential risk could not be avoided a priori. Interestingly, the indication of the pharmacogenetic study in Psychiatry was significantly different compared to that in Allergy, this may be due to the high number of patients with drug reactions in this later speciality.

We also observed a correlation between CYP2C9 PGx test results and reasons for referral. Specifically, the majority of patients with genotypes predicting intermediate metabolizer status were included due to adverse events.

This study also demonstrates the limitations of genotyping platforms and the necessity to follow-up on no-call results. No-calls often imply the presence of rare or novel SNPs or haplotypes. Adding newly discovered variants and haplotypes into databases such as PharmVar will enhance research and ultimately improve test platforms and test interpretation. In addition, we also provide examples of cost savings and emphasize that PGx information is saving costs at the time it is requested and has the potential to lower costs long term.

The majority of patients were referred from psychiatry which we attribute, at least in part, to the previous experience of physicians in this area [42,43]. Polypharmacy in psychiatric patients is common, and variability in drug response is routinely being observed [35,44]. Therefore, it was not surprising that nearly all patients with variants affecting CYP2D6 metabolic capacity were taking at least one drug metabolized by this enzyme. Since numerous clinically used drugs are metabolized by CYP2D6 (20% or more [45]) and given the wide range of interindividual variability [39,46], our findings corroborate the importance of *CYP2D6* genotype and its contribution to adverse events in psychiatric patients. Although most patients were normal metabolizers, about 13% were poor, intermediate, or ultrarapid metabolizers and, thus, at risk of problems with the medication prescribed. We would also like to highlight patients with eating disorders, particularly those poor metabolizers that all improved after PGx testing and intervention based on their test results.

## 5. Conclusions

Although the integration of PGx into precision medicine continues to grow, many challenges remain. One challenge is integrating PGx information with all other data to improve drug therapy while saving costs. To address this, we have developed and applied a PGx model, 5SPM, which demonstrates the utility of PGx. Experience of over ten years supports the approach of individualized drug therapy, especially in the setting of polypharmacy.

## Figures and Tables

**Table 1:** Demographics and medication request.

Variable	Value
PATIENTS	
Total number of caucasian patients included:	210
- Average age (range; years)	48 (9–91)
- Male: Female (%)	50.5: 49.5
PHARMACOGENETIC ANALYSIS REQUEST	
Application request (% of total)	
- Adverse events	47.1
- Poor response to treatment	19.0
- Others	33.9
Medical specialties applicants (% of total)	
- Psychiatry	
- Eating disorders unit	65.2
- Allergy	9.5
- Others (rheumatology, pediatric, oncology, neurology, pharmacy, hematology, infectious disease)	4.7
	20.6

**Table 2:** Top 25 medications involved in potential drug–gene interactions in study population.

Rank	Drug	Drug-Gene Interaction Counts
1	Omeprazole	53
2	Quetiapine	47
3	Olanzapine	42
4	Risperidone	37
5	Venlafaxine	34
6	Aripiprazole	33
7	Sertraline	33
8	Valproic Acid	24
9	Paracetamol/Acetaminophen	24
10	Clonazepam	20
11	Haloperidol	20
12	Clozapine	19
13	Fluoxetine	18
14	Alprazolam	15
15	Escitalopram	15
16	Methadone	15
17	Zolpidem	15
18	Atorvastatin	13
19	Diazepam	11
20	Cholecalciferol	9
21	Trazodone	9
22	Carbamazepine	8
23	Paroxetine	8
24	Rosuvastatin	8
25	Bupropion	7

**Table 3:** Top 24 medications involved in potential drug–drug interactions in study population.

Rank	Drug	Drug-Drug Interaction Counts
1	Omeprazole	141
2	Olanzapine	138
3	Quetiapine	113
4	Sertraline	100
5	Valproic Acid	83
6	Aripiprazole	80
7	Venlafaxine	70
8	Clozapine	68
9	Risperidone	65
10	Fluoxetine	62
11	Paracetamol/Acetaminophen	59
12	Clonazepam	59
13	Escitalopram	38
14	Methadone	35
15	Zolpidem	35
16	Haloperidol	32
17	Mirtazapine	31
18	Diazepam	27
19	Trazodone	27
20	Atorvastatin	25
21	Bupropion	24
22	Simvastatin	24
23	Cholecalciferol	23
24	Paroxetine	19

**Table 4:** Summary of variants tested.

Gene	Variants (SNPs)
CYP2C9	rs1799853 (CYP2C9*2)
	rs1057910 (CYP2C9*3)
CYP2C19	rs4244285 (CYP2C19*2)
	rs4986893 (CYP2C19*3)
	rs12248560 (CYP2C19*17)
CYP3A4	rs2740574 (CYP3A4*1b)
CYP3A5	rs776746 (CYP3A5*3)
ABCB1	rs1045642 (C3435T)
CYP2D6	rs1080985 (CYP2D6*2A)
	rs1065852 (CYP2D6*10 and *4)
	rs28371706 (CYP2D6*17, *40, *58 and *64)
	rs5030655 (CYP2D6*6)
	rs5030865 (CYP2D6*8 and *14)
	rs3892097 (CYP2D6*4)
	rs5030862 (CYP2D6*12)
	rs61736512 (CYP2D6*1, *1xN, *2xN, *3xN, *4xN, *6xN, *9x2, *10x2, *17x2, *29, *29x2, *35xN, *36xN, *41x2, *43xN, *45xN, *70, *107 and *149)
	rs28371725 (CYP2D6*41)
	rs35742686 (CYP2D6*3)
	rs5030656 (CYP2D6*9)
	rs16947 (CYP2D6*2)
	rs5030867 (CYP2D6*7)
CYP2B6	rs3745274 (CYP2B6*6)
CYP1A2	rs762551 (CYP1A2*1F)

## Data Availability

The data presented in this study are available on request from the corresponding author. The data are not publicly available due to privacy.

## Supplementary Materials

The following are available online at https://www.mdpi.com/article/10.3390/pharmaceutics14010160/s1, Figure S1: reasons for genetic analysis and clinical services, Figure S2: Main medical specialities applicants, Figure S3: Age box plot distribution in women and men, Figure S4: Top 10 drug-gene and drug-drug interactions.
