# Supplementary material for: Ten Years of Experience Support Pharmacogenetic Testing to Guide Individualized Drug Therapy
Source: Pharmaceutics. 2022 Jan 11;14(1):160. doi: 10.3390/pharmaceutics14010160 (PMC8779486; doi:10.3390/pharmaceutics14010160)
Supplement: Supplementary file 1 [file pharmaceutics-14-00160-s001.zip › pharmaceutics-1524552-supplementary.pdf]

Supplemenary Materials

Ten Years of Experience Support Pharmacogenetic Testing to Guide Individualized Drug Therapy

María Celsa Peña-Martín, Belén García-Berrocal, Almudena Sánchez-Martín, Elena Marcos-Vadillo, María Jesús García-Salgado, Santiago Sánchez, Carolina Lorenzo, David González-Parra, Francisco Sans, Manuel Franco, Andrea Gaedigk, María José Mateos-Sexmero, Catalina Sanz and María Isidoro-García

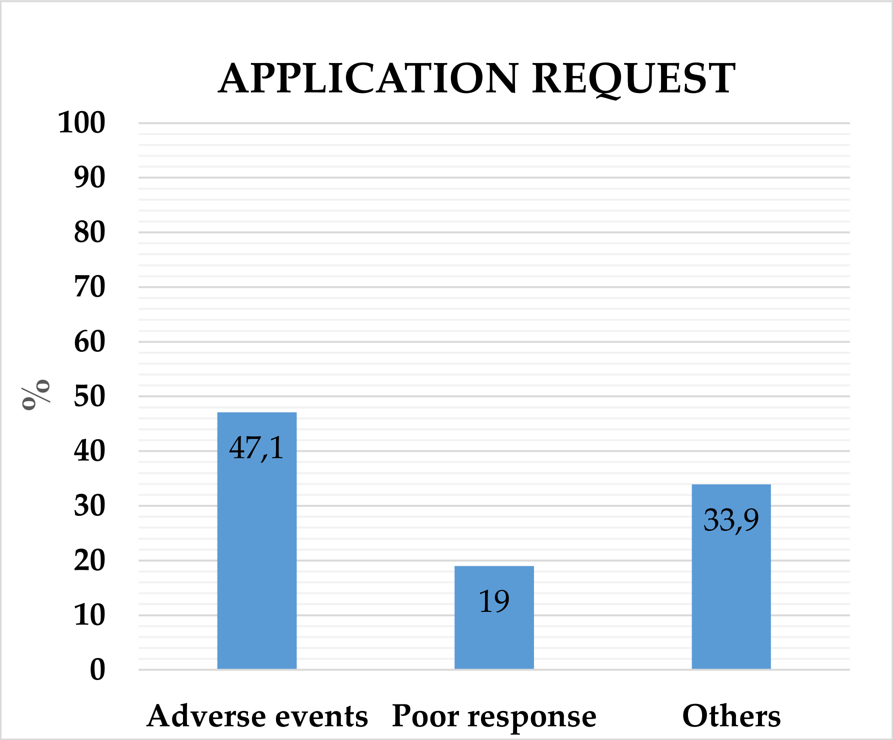

Figure S1. Reasons for genetic analysis and clinical services.

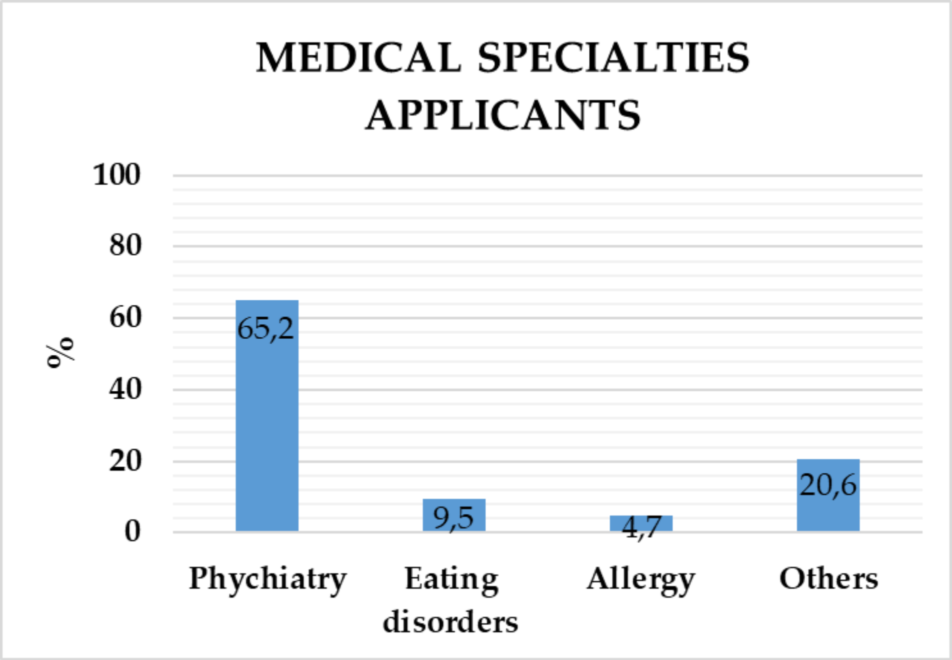

Figure S2. Main medical specialties applicants.

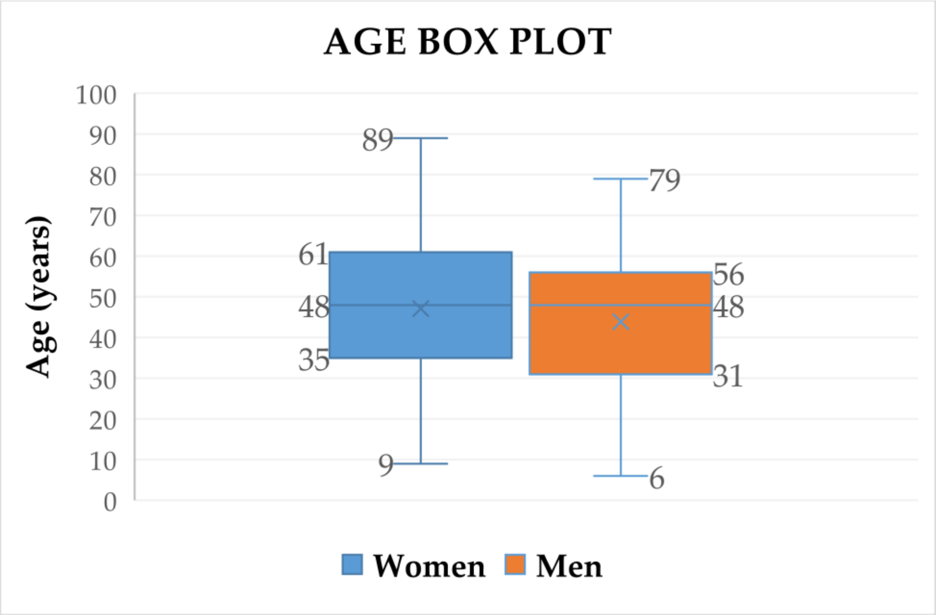

Figure S3. Age blox plot distribution in women and men.

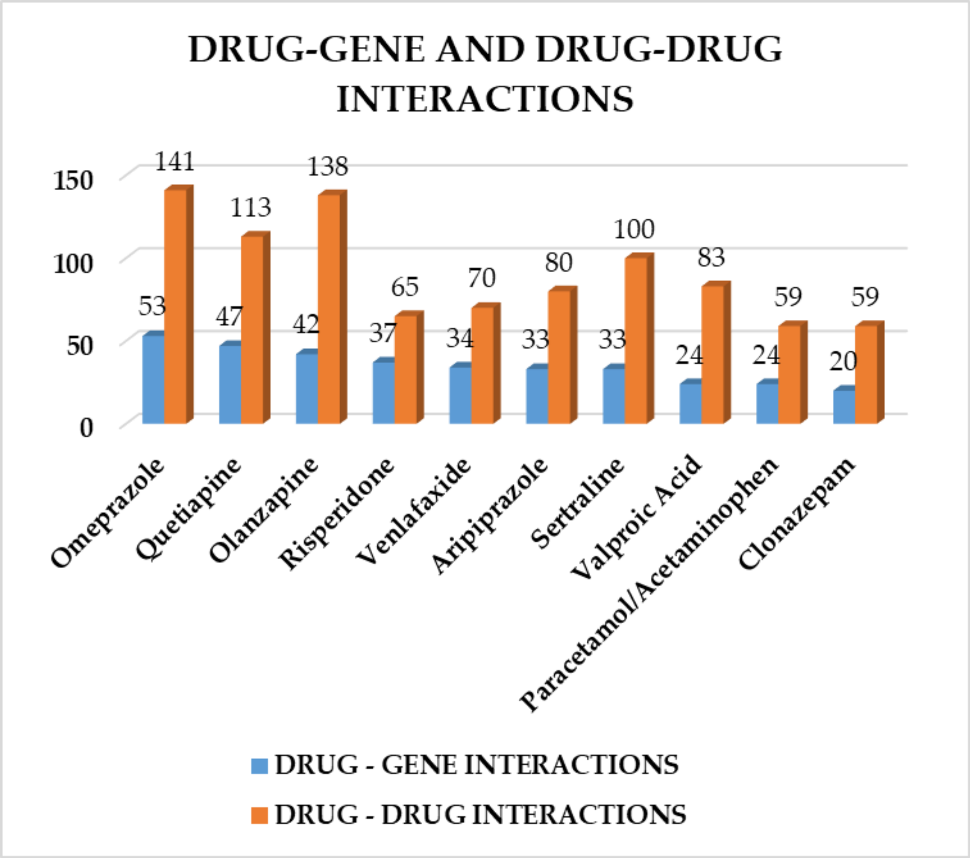

Figure S4. Top 10 Drug-gene and drug-drug interactions.
